# Supplementary figures and images for: Simulated trawling: Exhaustive swimming followed by extreme crowding as contributing reasons to variable fillet quality in trawl-caught Atlantic cod (Gadus morhua)
Source: PLoS One. 2020 Jun 18;15(6):e0234059. doi: 10.1371/journal.pone.0234059 (PMC7302710; doi:10.1371/journal.pone.0234059)

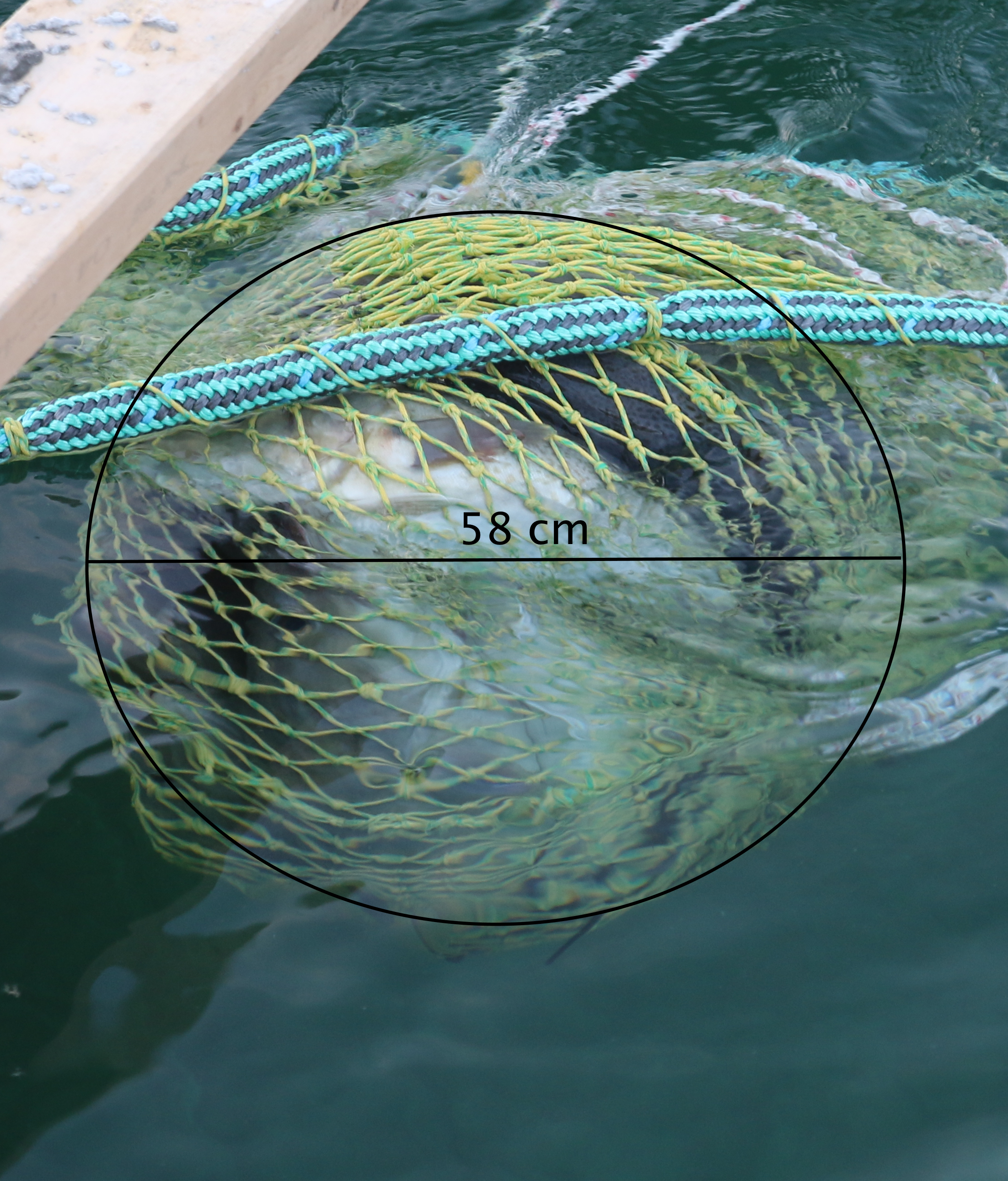

Supplement: S1 Fig — Image showing the extreme crowding of cod in the experimental cod-end. The shape of the closed cod-end resembled a sphere with diameter 58 cm. (TIF) [file pone.0234059.s001.tif]
