# Supplementary material for: Simulated trawling: Exhaustive swimming followed by extreme crowding as contributing reasons to variable fillet quality in trawl-caught Atlantic cod (Gadus morhua)
Source: PLoS One. 2020 Jun 18;15(6):e0234059. doi: 10.1371/journal.pone.0234059 (PMC7302710; doi:10.1371/journal.pone.0234059)

# Fig 1. Correlation between plasma cortisol and GSI

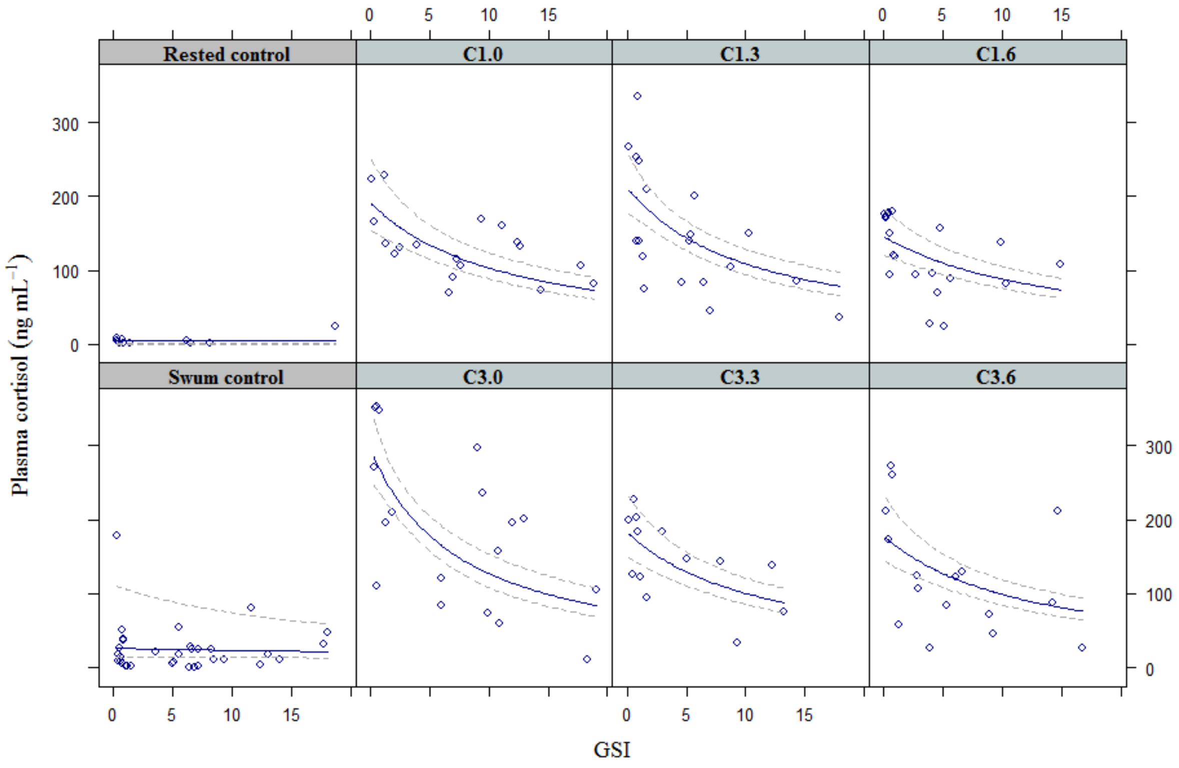


# Fig 2. Correlation between blood glucose and HSI

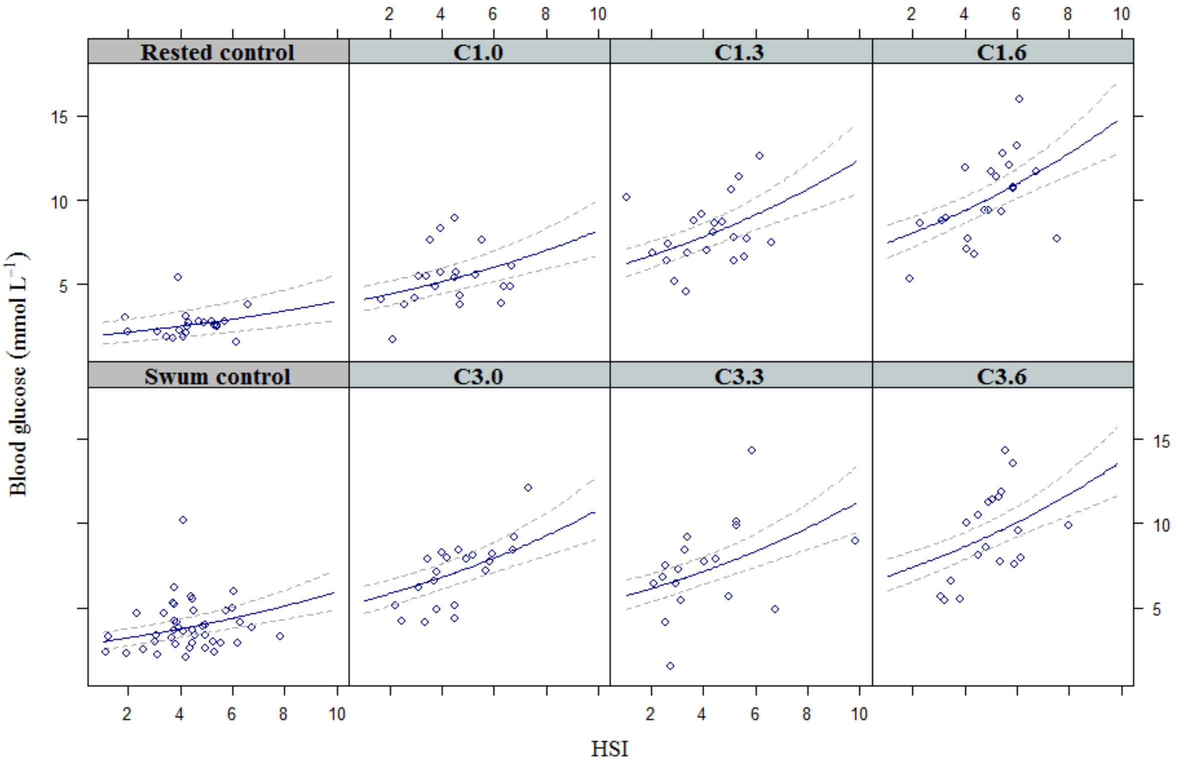

## Fig 3. Correlation between blood lactate and muscle pH.

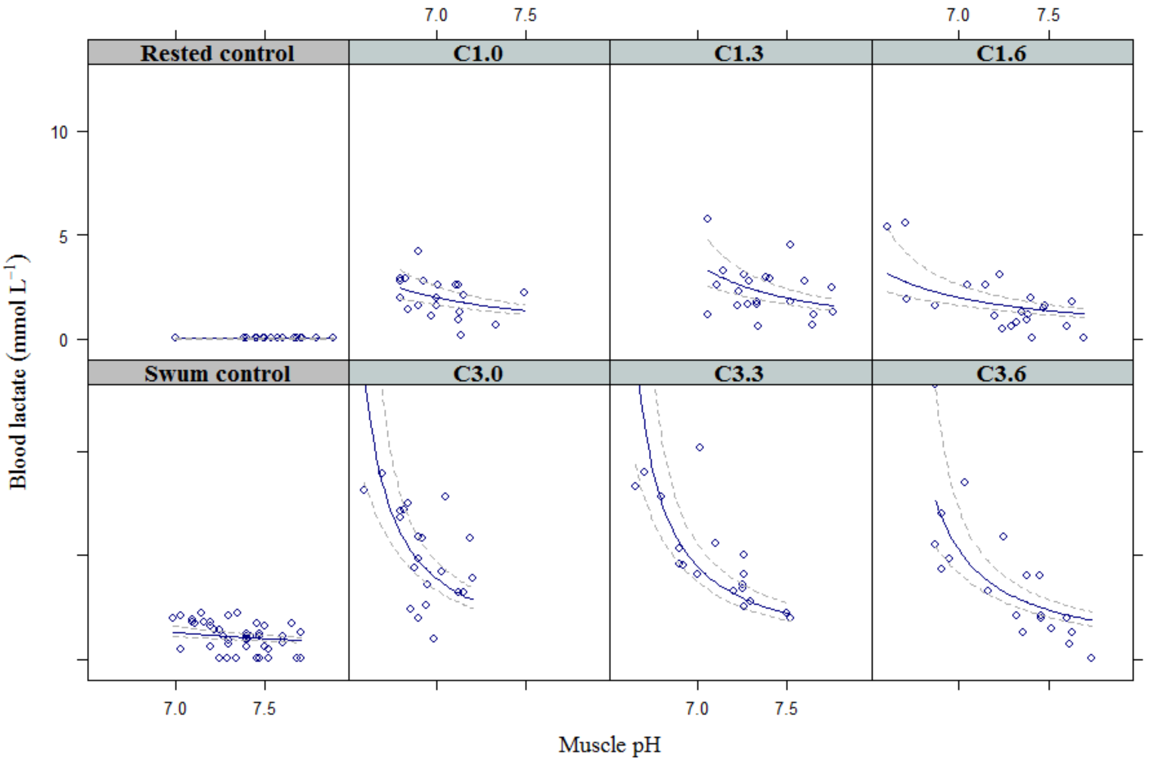


## Fig 4. Correlation between fillet redness and muscle haemoglobin

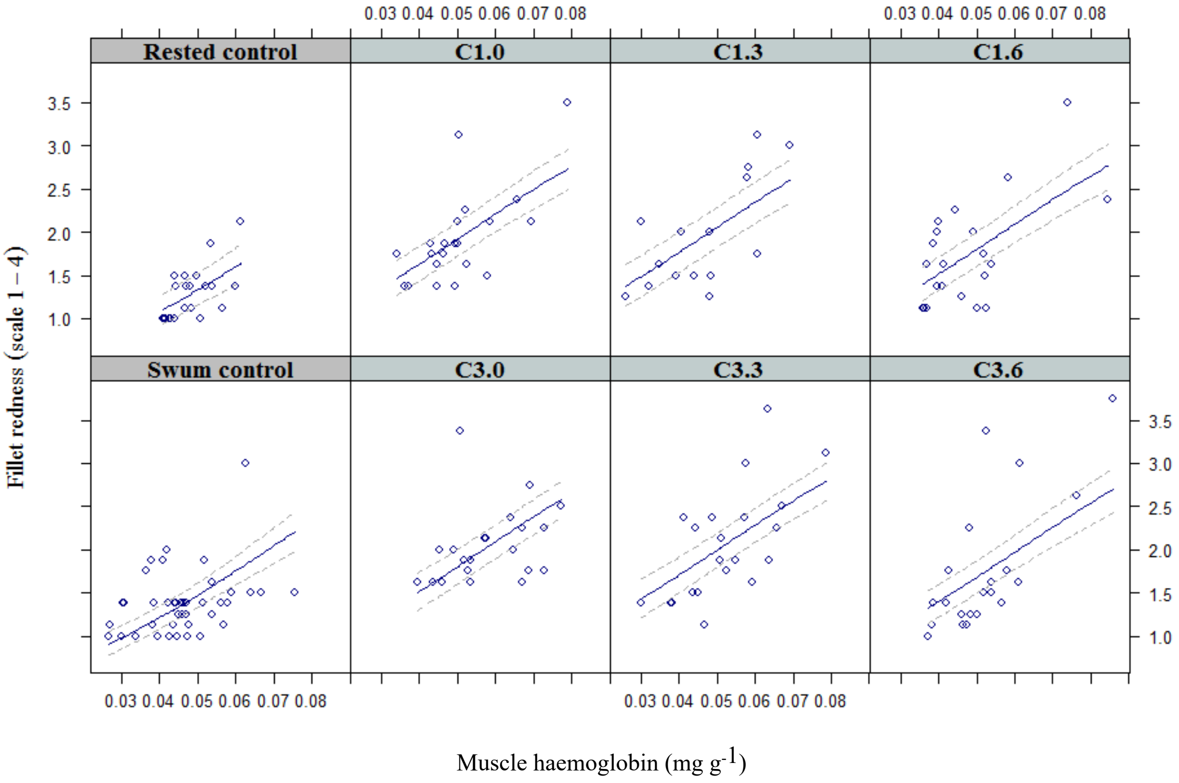

# Fig 5. Correlation between fillet redness and plasma cortisol

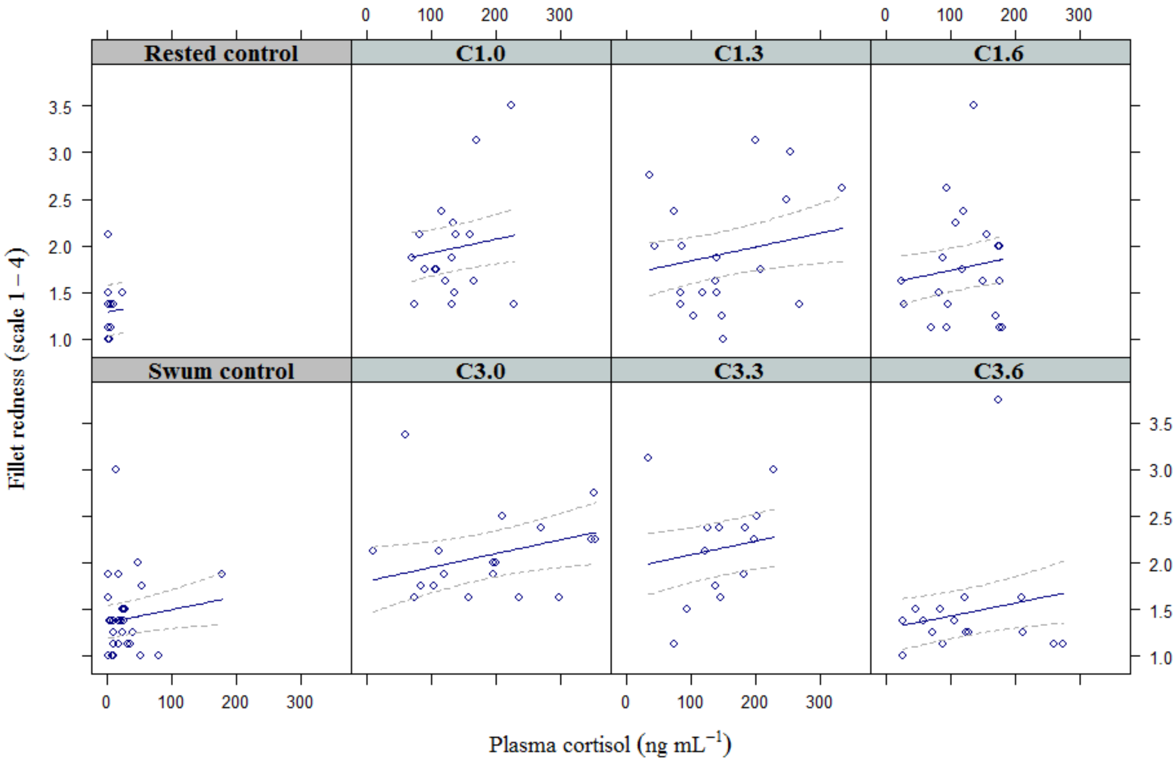


# Fig 6. Correlation between muscle haemoglobin and haematocrit

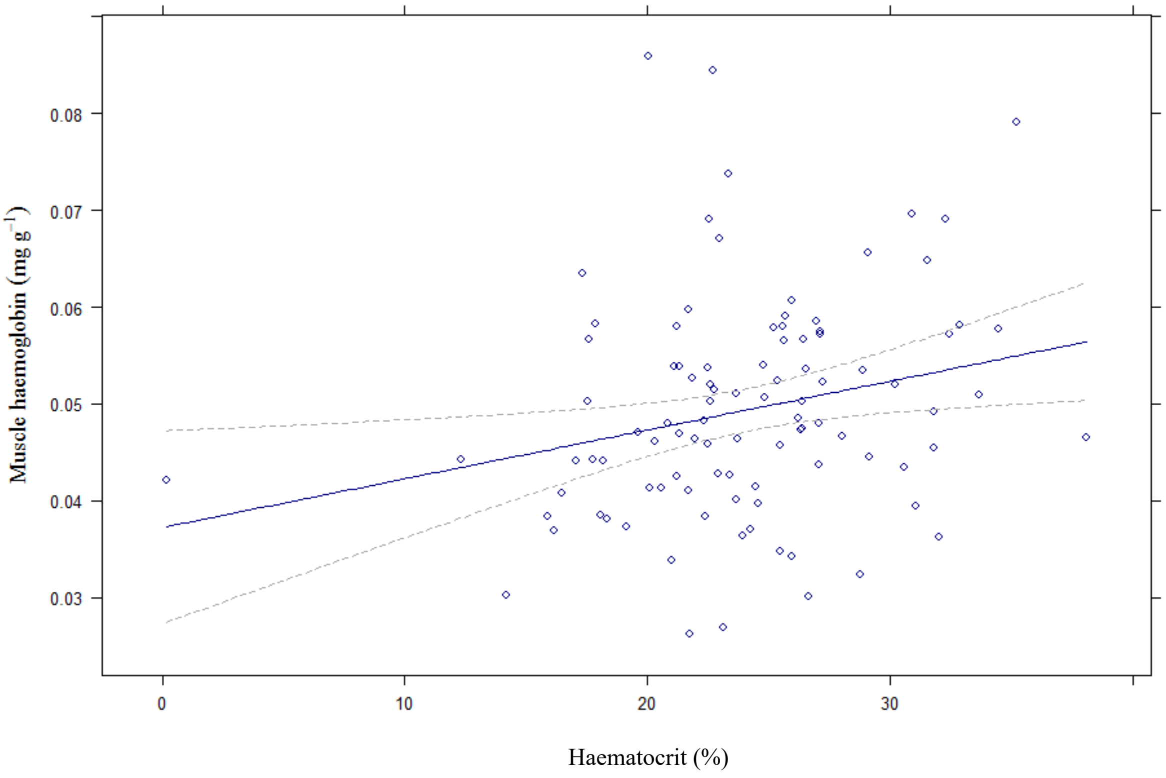

# Fig 7. Correlation between muscle haemoglobin and cortisol

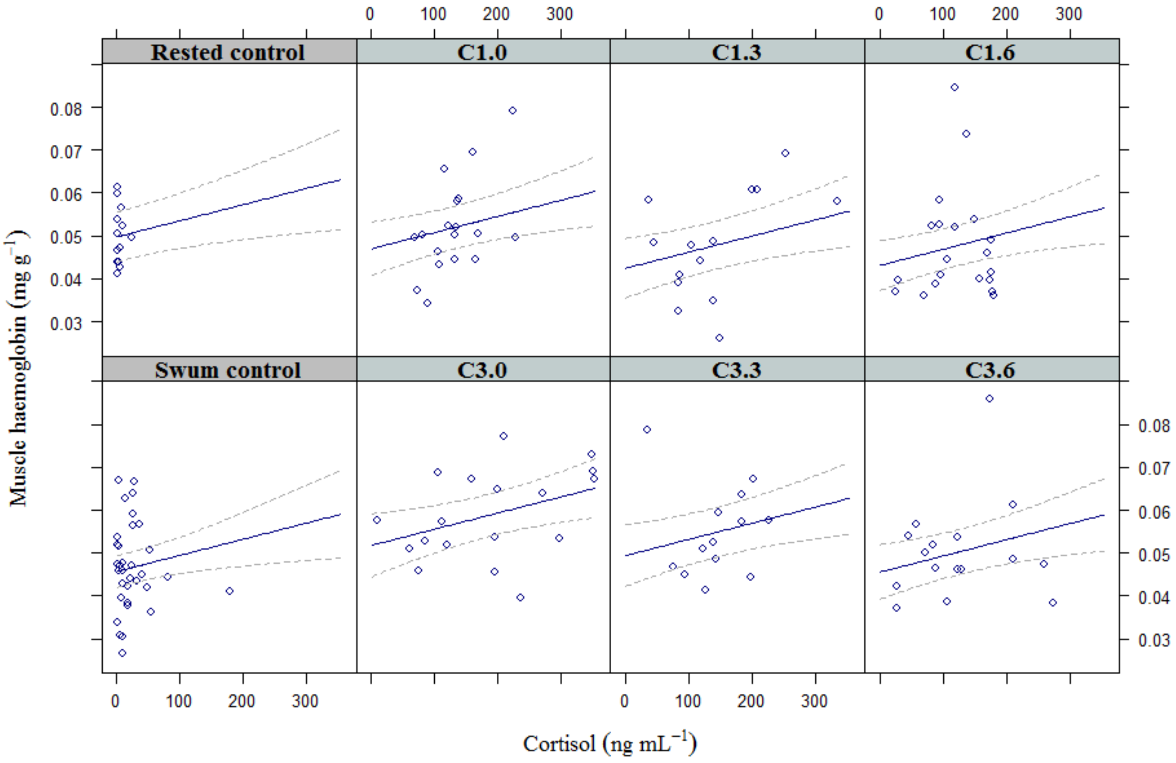

# Fig 8. Correlation between haematocrit and plasma cortisol

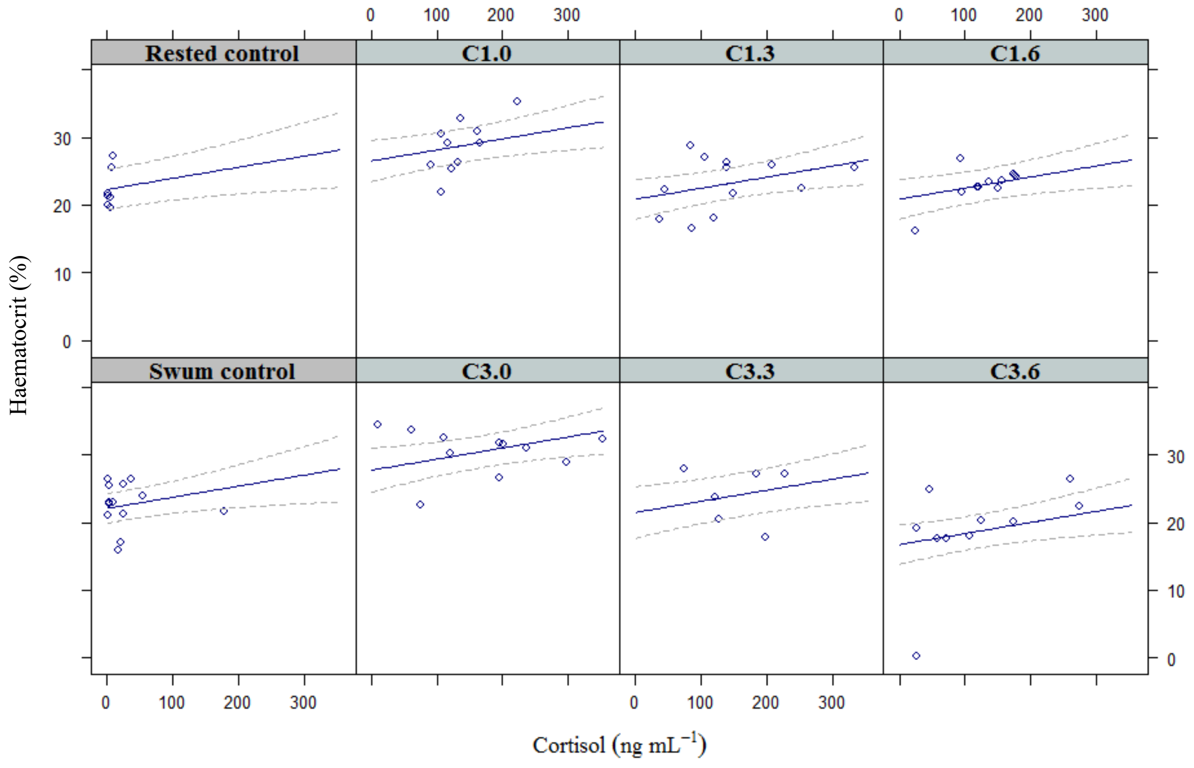

Supplement: S2 Fig — (PDF) [file pone.0234059.s002.pdf]
