# Supplementary material for: Simulated trawling: Exhaustive swimming followed by extreme crowding as contributing reasons to variable fillet quality in trawl-caught Atlantic cod (Gadus morhua)
Source: PLoS One. 2020 Jun 18;15(6):e0234059. doi: 10.1371/journal.pone.0234059 (PMC7302710; doi:10.1371/journal.pone.0234059)
Supplement: S1 Model — (PDF) [file pone.0234059.s003.pdf]

S3 Model details

Output from Generalized linear models

Cortisol

```
Call:
glm(formula = cort ~ treatment + gsi, family = gaussian(inverse),
    data = df)

Deviance Residuals:
    Min       1Q   Median       3Q      Max
-165.60   -23.93    -3.34    30.73   163.00

Coefficients:
                     Estimate Std. Error t value Pr(>|t|)
(Intercept)         2.237e-01  7.357e-01   0.304    0.762
treatmentpack.1.0  -2.184e-01  7.357e-01  -0.297    0.767
treatmentpack.1.3  -2.189e-01  7.357e-01  -0.298    0.766
treatmentpack.1.6  -2.168e-01  7.357e-01  -0.295    0.769
treatmentpack.3.0  -2.203e-01  7.357e-01  -0.299    0.765
treatmentpack.3.3  -2.182e-01  7.357e-01  -0.297    0.767
treatmentpack.3.6  -2.180e-01  7.357e-01  -0.296    0.767
treatments.control -1.855e-01  7.359e-01  -0.252    0.801
gsi                 4.471e-04  7.317e-05   6.111 9.17e-09 ***
---
Signif. codes:  0 ‘***’ 0.001 ‘**’ 0.01 ‘*’ 0.05 ‘.’ 0.1 ‘ ’ 1

(Dispersion parameter for gaussian family taken to be 2737.255)

    Null deviance: 1112242  on 149  degrees of freedom
Residual deviance:  385946  on 141  degrees of freedom
  (40 observations deleted due to missingness)
AIC: 1623.6

Number of Fisher Scoring iterations: 9
```

Analysis of Deviance Table

```
Model: gaussian, link: inverse

Response: cort

Terms added sequentially (first to last)


          Df Deviance Resid. Df Resid. Dev      F    Pr(>F)
NULL                        149    1112242
treatment  7   554006       142     558235 28.913 < 2.2e-16 ***
gsi        1   172290       141     385946 62.943 5.956e-13 ***
---
Signif. codes:  0 ‘***’ 0.001 ‘**’ 0.01 ‘*’ 0.05 ‘.’ 0.1 ‘ ’ 1


>
```

Glucose

```
Call:
glm(formula = glu ~ treatment + hsi, family = gaussian(log),
    data = df)

Deviance Residuals:
    Min       1Q   Median       3Q      Max
-4.9957  -1.1436  -0.0911   0.9309   6.4114
```

```
Coefficients:
                    Estimate Std. Error t value Pr(>|t|)    
(Intercept)          0.62007    0.16346   3.793 0.000205 ***
treatmentpack.1.0    0.71497    0.16867   4.239 3.63e-05 ***
treatmentpack.1.3    1.13090    0.15933   7.098 3.04e-11 ***
treatmentpack.1.6    1.31248    0.15660   8.381 1.66e-14 ***
treatmentpack.3.0    0.99200    0.16123   6.153 5.04e-09 ***
treatmentpack.3.3    1.04070    0.16217   6.417 1.26e-09 ***
treatmentpack.3.6    1.22771    0.15805   7.768 6.47e-13 ***
treatments.control   0.39689    0.16786   2.364 0.019157 *  
hsi                  0.07691    0.01334   5.764 3.64e-08 ***
---
Signif. codes:  0 ‘***’ 0.001 ‘**’ 0.01 ‘*’ 0.05 ‘.’ 0.1 ‘ ’ 1

(Dispersion parameter for gaussian family taken to be 3.330925)

    Null deviance: 1809.80  on 183  degrees of freedom
Residual deviance:  582.91  on 175  degrees of freedom
  (6 observations deleted due to missingness)
AIC: 754.34

Number of Fisher Scoring iterations: 5
```

Analysis of Deviance Table

```
Model: gaussian, link: log

Response: glu

Terms added sequentially (first to last)


          Df Deviance Resid. Df Resid. Dev      F    Pr(>F)    
NULL                        183    1809.80                     
treatment  7   1111.8       176     698.01 47.682 < 2.2e-16 ***
hsi        1    115.1       175     582.91 34.555  2.05e-08 ***
---
Signif. codes:  0 ‘***’ 0.001 ‘**’ 0.01 ‘*’ 0.05 ‘.’ 0.1 ‘ ’ 1
```

Lactate

```
Call:
glm(formula = lac ~ treatment + mpH, family = Gamma(inverse), 
    data = df)

Deviance Residuals: 
     Min        1Q    Median        3Q       Max  
-2.58366  -0.33619  -0.00047   0.27781   0.99015  

Coefficients:
                     Estimate Std. Error t value Pr(>|t|)    
(Intercept)          36.53743    4.37497   8.351 1.99e-14 ***
treatmentpack.1.0   -39.24542    4.35518  -9.011 3.46e-16 ***
treatmentpack.1.3   -39.46750    4.35499  -9.063 2.51e-16 ***
treatmentpack.1.6   -39.24458    4.35523  -9.011 3.47e-16 ***
treatmentpack.3.0   -39.48641    4.35498  -9.067 2.44e-16 ***
treatmentpack.3.3   -39.51815    4.35497  -9.074 2.34e-16 ***
treatmentpack.3.6   -39.55227    4.35491  -9.082 2.22e-16 ***
treatments.control  -38.97400    4.35532  -8.949 5.11e-16 ***
mpH                   0.45794    0.05559   8.238 3.96e-14 ***
---
Signif. codes:  0 ‘***’ 0.001 ‘**’ 0.01 ‘*’ 0.05 ‘.’ 0.1 ‘ ’ 1

(Dispersion parameter for Gamma family taken to be 0.2488991)

    Null deviance: 318.337  on 183  degrees of freedom
Residual deviance:  95.924  on 175  degrees of freedom
```

```
  (6 observations deleted due to missingness)
AIC: 442.75

Number of Fisher Scoring iterations: 5
```

Sensory evaluation of redness with muscle haemoglobin

```
Call:
glm(formula = (sens + 1)/4 ~ treatment + mbr, family = quasibinomial(), 
    data = df)

Deviance Residuals: 
     Min        1Q    Median        3Q       Max  
-0.39348  -0.15090  -0.03632   0.10519   0.84174  

Coefficients:
                   Estimate Std. Error t value Pr(>|t|)    
(Intercept)         -2.1613     0.1912 -11.302  < 2e-16 ***
treatmentpack.1.0    0.6153     0.1465   4.200 4.25e-05 ***
treatmentpack.1.3    0.7535     0.1601   4.708 5.10e-06 ***
treatmentpack.1.6    0.4951     0.1472   3.363 0.000948 ***
treatmentpack.3.0    0.4948     0.1497   3.306 0.001151 ** 
treatmentpack.3.3    0.6906     0.1469   4.701 5.25e-06 ***
treatmentpack.3.6    0.3723     0.1459   2.552 0.011566 *  
treatments.control   0.1552     0.1307   1.188 0.236567    
mbr                 29.2960     3.2643   8.975 4.49e-16 ***
---
Signif. codes:  0 ‘***’ 0.001 ‘**’ 0.01 ‘*’ 0.05 ‘.’ 0.1 ‘ ’ 1

(Dispersion parameter for quasibinomial family taken to be 0.0516939)

    Null deviance: 17.5746  on 182  degrees of freedom
Residual deviance:  9.3824  on 174  degrees of freedom
  (7 observations deleted due to missingness)
AIC: NA

Number of Fisher Scoring iterations: 4
```

Analysis of Deviance Table

```
Model: quasibinomial, link: logit

Response: (sens + 1)/4

Terms added sequentially (first to last)


          Df Deviance Resid. Df Resid. Dev      F    Pr(>F)    
NULL                        182    17.5746                     
treatment  7   3.8829       175    13.6917 10.731 3.288e-11 ***
mbr        1   4.3093       174     9.3824 83.362 < 2.2e-16 ***
---
Signif. codes:  0 ‘***’ 0.001 ‘**’ 0.01 ‘*’ 0.05 ‘.’ 0.1 ‘ ’ 1
```

Sensory evaluation of redness with plasma cortisol

```
Call:
glm(formula = (sens + 1)/4 ~ treatment + cort, family = quasibinomial, 
    data = df)

Deviance Residuals: 
     Min        1Q    Median        3Q       Max  
-0.47022  -0.16657  -0.03464   0.09097   1.17908  
```

```
Coefficients:
                      Estimate Std. Error t value Pr(>|t|)    
(Intercept)         -0.7397988  0.1585117  -4.667 7.07e-06 ***
treatmentpack.1.0    0.5154926  0.2228334   2.313  0.02216 *  
treatmentpack.1.3    0.4308118  0.2269462   1.898  0.05971 .  
treatmentpack.1.6    0.3279003  0.2163676   1.515  0.13191    
treatmentpack.3.0    0.5452524  0.2421873   2.251  0.02592 *  
treatmentpack.3.3    0.6802684  0.2398435   2.836  0.00524 ** 
treatmentpack.3.6    0.0074835  0.2289634   0.033  0.97397    
treatments.control   0.0812914  0.1875940   0.433  0.66544    
cort                 0.0014818  0.0007247   2.045  0.04275 *  
---
Signif. codes:  0 ‘***’ 0.001 ‘**’ 0.01 ‘*’ 0.05 ‘.’ 0.1 ‘ ’ 1

(Dispersion parameter for quasibinomial family taken to be 0.07156997)

    Null deviance: 14.116  on 148  degrees of freedom
Residual deviance: 10.392  on 140  degrees of freedom
  (41 observations deleted due to missingness)
AIC: NA

Number of Fisher Scoring iterations: 3
```

Analysis of Deviance Table

```
Model: quasibinomial, link: logit

Response: (sens + 1)/4

Terms added sequentially (first to last)


          Df Deviance Resid. Df Resid. Dev      F    Pr(>F)    
NULL                        148     14.116                     
treatment  7   3.4236       141     10.692 6.8338 5.525e-07 ***
cort       1   0.2999       140     10.392 4.1901   0.04253 *  
---
Signif. codes:  0 ‘***’ 0.001 ‘**’ 0.01 ‘*’ 0.05 ‘.’ 0.1 ‘ ’ 1
```

Muscle haemoglobin modelled with haematocrit

```
Call:  glm(formula = (mbr) ~ hct, family = gaussian(), data = df)


Deviance Residuals: 
      Min          1Q      Median          3Q         Max  
-0.022056  -0.007248  -0.000999   0.006001   0.038645  

Coefficients:
             Estimate Std. Error t value Pr(>|t|)    
(Intercept) 0.0372313  0.0050855   7.321 7.22e-11 ***
hct         0.0005040  0.0002062   2.444   0.0163 *  
---
Signif. codes:  0 ‘***’ 0.001 ‘**’ 0.01 ‘*’ 0.05 ‘.’ 0.1 ‘ ’ 1

(Dispersion parameter for gaussian family taken to be 0.0001254236)

    Null deviance: 0.012915  on 98  degrees of freedom
Residual deviance: 0.012166  on 97  degrees of freedom
  (91 observations deleted due to missingness)
AIC: -604.47

Number of Fisher Scoring iterations: 2
```

Analysis of Deviance Table

```
Model: gaussian, link: identity
```

```
Response: (mbr)

Terms added sequentially (first to last)


     Df   Deviance Resid. Df Resid. Dev      F  Pr(>F)  
NULL                      98   0.012915                 
hct   1 0.00074912        97   0.012166 5.9727 0.01634 *
---
Signif. codes:  0 ‘***’ 0.001 ‘**’ 0.01 ‘*’ 0.05 ‘.’ 0.1 ‘ ’ 1
```

Muscle haemoglobin without haematocrit

```
Call:
glm(formula = mbr ~ treatment + cort, family = gaussian(), data = df)

Deviance Residuals: 
      Min         1Q     Median         3Q        Max  
-0.021899  -0.007157  -0.001759   0.005926   0.036879  

Coefficients:
                     Estimate Std. Error t value Pr(>|t|)    
(Intercept)         4.977e-02  2.970e-03  16.761   <2e-16 ***
treatmentpack.1.0  -2.771e-03  4.329e-03  -0.640   0.5231    
treatmentpack.1.3  -7.339e-03  4.591e-03  -1.598   0.1123    
treatmentpack.1.6  -6.657e-03  4.182e-03  -1.592   0.1138    
treatmentpack.3.0   2.019e-03  4.741e-03   0.426   0.6709    
treatmentpack.3.3  -3.577e-04  4.677e-03  -0.076   0.9391    
treatmentpack.3.6  -4.212e-03  4.378e-03  -0.962   0.3377    
treatments.control -4.062e-03  3.518e-03  -1.155   0.2503    
cort                3.767e-05  1.473e-05   2.557   0.0117 *  
---
Signif. codes:  0 ‘***’ 0.001 ‘**’ 0.01 ‘*’ 0.05 ‘.’ 0.1 ‘ ’ 1

(Dispersion parameter for gaussian family taken to be 0.0001145962)

    Null deviance: 0.018644  on 144  degrees of freedom
Residual deviance: 0.015585  on 136  degrees of freedom
  (45 observations deleted due to missingness)
AIC: -893.54

Number of Fisher Scoring iterations: 2
```

Analysis of Deviance Table

```
Model: gaussian, link: identity

Response: mbr

Terms added sequentially (first to last)


          Df   Deviance Resid. Df Resid. Dev      F   Pr(>F)   
NULL                          144   0.018644                   
treatment  7 0.00230938       137   0.016334 2.8789 0.007785 **
cort       1 0.00074931       136   0.015585 6.5387 0.011652 * 
---
Signif. codes:  0 ‘***’ 0.001 ‘**’ 0.01 ‘*’ 0.05 ‘.’ 0.1 ‘ ’ 1
```

Haemotocrit

```
Call:
glm(formula = (hct) ~ treatment + cort, family = gaussian(), 
    data = df)

Deviance Residuals: 
```

```
     Min        1Q    Median        3Q       Max
-17.0103   -1.6971    0.3383    2.3208    7.3038

Coefficients:
                    Estimate Std. Error t value Pr(>|t|)
(Intercept)        22.326168   1.511959  14.766   <2e-16 ***
treatmentpack.1.0   4.194346   2.165753   1.937   0.0568 .
treatmentpack.1.3  -1.464930   2.119666  -0.691   0.4917
treatmentpack.1.6  -1.446498   2.112939  -0.685   0.4958
treatmentpack.3.0   5.400214   2.233996   2.417   0.0182 *
treatmentpack.3.3  -0.838822   2.452710  -0.342   0.7334
treatmentpack.3.6  -5.592258   2.114374  -2.645   0.0101 *
treatments.control -0.234210   1.882553  -0.124   0.9013
cort                0.016383   0.006802   2.409   0.0186 *
---
Signif. codes:  0 ‘***’ 0.001 ‘**’ 0.01 ‘*’ 0.05 ‘.’ 0.1 ‘ ’ 1

(Dispersion parameter for gaussian family taken to be 15.99685)

    Null deviance: 2265.3  on 79  degrees of freedom
Residual deviance: 1135.8  on 71  degrees of freedom
  (110 observations deleted due to missingness)
AIC: 459.27

Number of Fisher Scoring iterations: 2
```

Analysis of Deviance Table

```
Model: gaussian, link: identity

Response: (hct)

Terms added sequentially (first to last)


          Df Deviance Resid. Df Resid. Dev     F    Pr(>F)
NULL                         79     2265.3
treatment  7  1036.69        72     1228.6 9.258 4.331e-08 ***
cort       1    92.81        71     1135.8 5.802   0.01861 *
---
Signif. codes:  0 ‘***’ 0.001 ‘**’ 0.01 ‘*’ 0.05 ‘.’ 0.1 ‘ ’ 1
```

Muscle pH

```
Call:
glm(formula = pH ~ adj.hrs * treatment + I(adj.hrs^2) * treatment,
    family = gaussian(), data = rigor)

Deviance Residuals:
     Min        1Q    Median        3Q       Max
-0.73550  -0.15952  -0.01063   0.16668   0.84081

Coefficients:
                                  Estimate Std. Error t value Pr(>|t|)
(Intercept)                      7.571e+00  5.091e-02 148.715  < 2e-16 ***
adj.hrs                         -2.988e-02  3.150e-03  -9.486  < 2e-16 ***
treatmentpack.1.0               -5.497e-01  7.239e-02  -7.594 6.32e-14 ***
treatmentpack.1.3               -1.744e-01  7.192e-02  -2.425  0.01548 *
treatmentpack.1.6               -2.957e-01  7.130e-02  -4.147 3.61e-05 ***
treatmentpack.3.0               -5.178e-01  7.236e-02  -7.155 1.47e-12 ***
treatmentpack.3.3               -3.065e-01  7.441e-02  -4.120 4.06e-05 ***
treatmentpack.3.6               -9.082e-02  7.252e-02  -1.252  0.21073
treatments.control              -1.370e-01  6.119e-02  -2.239  0.02534 *
I(adj.hrs^2)                     2.126e-04  4.425e-05   4.804 1.75e-06 ***
adj.hrs:treatmentpack.1.0        1.731e-02  4.334e-03   3.993 6.93e-05 ***
adj.hrs:treatmentpack.1.3        1.052e-02  4.435e-03   2.371  0.01788 *
adj.hrs:treatmentpack.1.6        1.479e-02  4.547e-03   3.252  0.00118 **
```

```
adj.hrs:treatmentpack.3.0        5.229e-03  4.310e-03   1.213  0.22530
adj.hrs:treatmentpack.3.3       -2.206e-04  4.533e-03  -0.049  0.96120
adj.hrs:treatmentpack.3.6       -5.302e-03  4.606e-03  -1.151  0.24994
adj.hrs:treatments.control       4.093e-03  3.700e-03   1.106  0.26884
treatmentpack.1.0:I(adj.hrs^2)  -1.362e-04  5.827e-05  -2.337  0.01960 *
treatmentpack.1.3:I(adj.hrs^2)  -1.161e-04  6.060e-05  -1.915  0.05568 .
treatmentpack.1.6:I(adj.hrs^2)  -1.795e-04  6.343e-05  -2.830  0.00473 **
treatmentpack.3.0:I(adj.hrs^2)   4.963e-06  5.844e-05   0.085  0.93234
treatmentpack.3.3:I(adj.hrs^2)   8.367e-05  6.195e-05   1.350  0.17714
treatmentpack.3.6:I(adj.hrs^2)   1.035e-04  6.448e-05   1.606  0.10865
treatments.control:I(adj.hrs^2) -5.230e-05  5.072e-05  -1.031  0.30274
---
Signif. codes:  0 ‘***’ 0.001 ‘**’ 0.01 ‘*’ 0.05 ‘.’ 0.1 ‘ ’ 1

(Dispersion parameter for gaussian family taken to be 0.06071145)

    Null deviance: 185.904  on 1198  degrees of freedom
Residual deviance:  71.336  on 1175  degrees of freedom
  (265 observations deleted due to missingness)
AIC: 69.225

Number of Fisher Scoring iterations: 2
```

Analysis of Deviance Table

```
Model: gaussian, link: identity

Response: pH

Terms added sequentially (first to last)


                       Df Deviance Resid. Df Resid. Dev         F    Pr(>F)
NULL                                    1198    185.904
adj.hrs                 1   86.570      1197     99.334 1425.9288 < 2.2e-16
***
treatment               7   11.190      1190     88.144   26.3295 < 2.2e-16
***
I(adj.hrs^2)            1   10.265      1189     77.880  169.0742 < 2.2e-16
***
adj.hrs:treatment       7    4.168      1182     73.712    9.8070 6.355e-12
***
treatment:I(adj.hrs^2)  7    2.376      1175     71.336    5.5904 2.353e-06
***
---
Signif. codes:  0 ‘***’ 0.001 ‘**’ 0.01 ‘*’ 0.05 ‘.’ 0.1 ‘ ’ 1
```
