# Supplementary material for: Simulated trawling: Exhaustive swimming followed by extreme crowding as contributing reasons to variable fillet quality in trawl-caught Atlantic cod (Gadus morhua)
Source: PLoS One. 2020 Jun 18;15(6):e0234059. doi: 10.1371/journal.pone.0234059 (PMC7302710; doi:10.1371/journal.pone.0234059)
Supplement: Attachment — Submitted filename: Answer to reviewers.pdf [file pone.0234059.s004.pdf]

Dear Editors

We thank the reviewers for their generous comments on the manuscript and have edited the manuscript to address their concerns.

We have made major changes to the description of experimental protocol and the discussion have been revised substantially.

We believe that the manuscript is now suitable for publication.

Dr. Ragnhild A. Svalheim
Scientific Researcher, Seafood Industry, Nofima AS

On behalf of all authors.

Reviewer #1:

The experimental protocol used to exhausted and crowded the fish is relevant and well-conceived. The metabolic state of the fish has been well investigated with blood lactate, plasma glucose, plasma cortisol and muscle pH. The flesh quality analysis is more restricted but focused on flesh colour and haemoglobin content as the main flesh quality indicator for consumer.

However the choice of the control groups is not optimal, indeed there's no group of non exhausted fish but only crowded, so it's not possible in the effect observed to analysed the impact of crowding only, such result should be interesting as the same authors found previously that exhaustive swimming alone cannot reproduce the effect of trawling, so what the impact of crowding only ? Thank you for this comment. Indeed, we had this discussion when designing the experiment and fully agree that this would be an interesting group to study. However, as one of or main objective was to simulate trawling, the "crowded only" group was left out of this. We also

figured that the fight to get the large rested fish into the small cod-end at the extreme densities used in the present study, probably would cause a stress response large enough that we still not would have analysed the impact of only crowding, but rather the impact of physical struggle and air exposure together with the effect of crowding.

The water quality monitoring during crowding appear very limited with only a dissolved oxygen measurement. Carbon dioxide and ammonia measurement should be also interesting as the authors failed to explained the anaerobic status of the fish with only dissolved oxygen data.
Thank you for this comment. We agree that these parameters would have strengthened the study.

Some additional data on fish rearing is necessary, indeed the fish were captured in May 2014 with a mean body weight of 3.5 kg and used 9 months later in February, in the table 1 the mean body weight of the fish is still 3.5 kg, does it mean that fish didn't grew in 9 months ? Add data on the growth and the mortality of the fish during this period.
Thank you for point this out. Biological data of the fish was obtained at each sampling day in is therefore the weight/length of the fish 10 months after capture.

In the statistical analysis line 245 "the data were strictly bound between 1 and 4" but line 204 "a score from 0 to 2..." please indicate the scale used.
Thanking you for pointing this out, it has now been corrected to the correct scale.

Minor revision:
Line 85: Add muscle haemoglobin in the measurements.
Haemoglobin has been added
Lines 107 in the table 1 for the length SD please homogenize the number of digits in accordance to the accuracy of the measure for example 74±7 instead of 74±6.61.
The table is updated
Line 177 "on the left side" instead of "on the aleft side"

This has been corrected

Reviewer 2

1

PONE-D-18-20898 – "Simulated trawling…"

Related to introduction and discussion parts, an incomplete review of literature is given. Atlantic cod is a widely studied animal and several important studies are omitted. In line with this, the authors discussion of results is not always correct. One gets the impression that selected research have been included to support the present findings. It should be remembered that it is the authors duty to provide a balanced view of literature, and from that, try to extract the current status of knowledge.

• The weakest part of the ms is the discussion chapter. It might as well be deleted and entirely re-written.
• Due to incomplete study of literature, some "doubtful" issues are discussed as there are several omitted papers showing contradictory results.
• This is a study of live fish and effect on fillet color. Key issues should be stress physiology and recovery and less on postmortem issues except from fillet color. Literature and discussion should reflect just that.
• Data do not always support conclusions

On the basis of these comments we decided to withdraw the original manuscript and to substantially revise the discussion and include a new paragraph where we discuss negative results from other studies.

Title: a new title should be provided. Avoid "exhaustive" and "fillet quality" (see below)

Abstract

L28: delete "size"¨

Size is deleted

L29: "…kg m-3…" Corrected

L31: delete "quickly" deleted

L34: it is not correct to speak of "drop in muscle pH" as reduction of quality (it inevitably occurs in all fish regardless of treatment, see below). Drop has been replaced with "lower initial"

Introduction

For readers not familiar with trawl fisheries, this chapter should provide some background info about commercial trawling of cod, like trawling procedures, typical catch sizes, speeds, etc and from that explain why it was necessary to study stress and crowding in a lab scale.

Thank you for this comment. We have included a short paragraph on trawls.

L53: delete "fatiguing"'

Deleted

L56-59: What does "quality" mean here? In this ms, the use of the word "quality" should be avoided. Remember that "flesh quality" is a rather complex issue where many variables will collectively constitute the quality of a muscle-based foodstuff. Hence, you did NOT measure quality here. Instead, as in this case, be specific of which quality parameter(s) you measured (color, coagulated blood). Otherwise, your statements may be considered overinterpreted and misleading. For example, a fillet may have some discoloration, but when all other quality parameters are taken into account, the fillet can still be of relatively good quality.

We appreciate your comment and agree that the term quality is incorrectly used in the manuscript. We have now replaced "quality" with haemoglobin or redness.

L63-65: Delete references (other fish species) and use readily available references on Atlantic cod instead. Generally, cod quality is less affected by stress compared with more active fish species. From that, you will see you will need to modify your statements. Also, cod fillet color is not always affected by stress (see literature).

L69: replace "will be" with "may be" since this is not always the case.

ok

L78: "quality" or a few variables? Were the quality improvements significant for processors and consumers?

"Quality" has been replaced with "fillet colour"

Materials and Methods

L95: "…group and treatment means…" should be better defined here

This sentence has been moved and altered after suggestion from reviewer 1. It now at the end of the paragraph to avoid confusion in difference between weight at capture day (not presented) and sampling day.

L96: transport duration?

12 hours, this is now included in the manuscript

L98: were the fish fed during this period?

No, during the three weeks the fish were in Nordvågen , they were not fed. This is now made clear in the manuscript.

L105: "…1.2 m/s…"

This is corrected

L106: among groups?

Yes. This is corrected

Table 1: is it the SDs that are shown? Length-delete decimals for SDs, i.e. 74 ± 7 etc. HIS- the number of decimals should be the same in all cases

Thank you for pointing this out. The table is corrected.

L109: "…row…" vs column?

Column indeed. This is corrected.

L143-145: what is the timeline here? If sampling of ALL fish (n=7) was done after 20 min x 28 = 560 min this will be equal to 560 min of recovery. Is my understanding of sampling correct?

If so, you do not have "swum control fish". Instead, you will then have more or less recovered fish (see also below and refer to your data on these fish).

There seems to be a misunderstanding regarding the experimental set up. In the updated manuscript we have attempted to describe the experiment more clearly. In addition, we have included a flow-chart illustrating the different stages of the trials. Hopefully, this change will explain the procedure in more understandable manner.

Firstly, all 28 fish were swum as a group simultaneously in a large swim tunnel. When max. water velocity was reached after 20 minutes, the fish were not able to swim for longer than max 15 minutes in the tunnel, meaning that the last fish in the retention chamber had at most swum 15 longer than the first one. Secondly, the water velocity in the retention chamber was higher than in the recuperation chamber (0.8 ms^{-1} versus 0.1-0.2 ms^{-1}) and therefore, swimming in the retention chamber would be considered exercise.

Regarding the swum control group, a pre-randomized list based on the order of exhaustion made separately for each run was used to select fish for the swum control. As a fish destined for the swum control group became exhausted and entered the retention chamber, it was immediately netted out and sampled. We have now included a clearer description of this in the manuscript.

Fig. 1 – two sentences which basically say the same thing. Merge to one sentence.

Fig 1 is now fig 2. There is now only one sentence describing this figure.

L167: "…natural water temperature…" ???; How do you know the water was oxygen-saturated (=100% DO)?

Water temperature is now included (see Table 1).

L168: "…checked…" How?

Visual inspection. This is now included in the manuscript.

L199: Fillet redness. This parameter is not clearly described causing confusion. The reviewer was unable to fully assess your findings since: (a) the scale 0-2 described (L204-205) is not the same as you used in the results section where the scale is 1-4! (b) was it only the overall color of fillets you evaluated? Was residual blood in veins and blood spots not taken into account?

We have tried to clarify this better in the text.

L211: "…model VNIR-640…". City? Added

L214: Skjelvareid et al. (14)?

This har been corrected.

L228-229: mM = mmol/l (not mM/l) This is now corrected

Results

L251: "…kg m-3…" Ok

L252: "…above 95 %...", are DO data shown somewhere?

DO is only presented in once sentence in the results, as it stayed at 100 % throughout the experiment. Lowest registered DO was 95 % and we do not this to have an impact the results.

L254: "…for 3 hours 18 %...", should that be 5 % (see Table S1)?

18 % is the average of the three runs. Mortality per run is now included in Table 1.

L276: Rested OK

L277: Here it is stated that swum control fish are sampled IMMEDIATELY after exhaustive exercise! This is very confusing! I suppose this means after the last fish was exercised?

Please see point L143-145.

L279: Even though CIs are shown in the figure, p-values should be indicated, where appropriate, above bars. Also, add "n = 7".

The bars indicate the combines results from the triplicates, leading up to N = 21. This is now included in figure text.

We believe, that in studies such as the present, testing under p-values becomes too simplistic and that the distinction between "significant" and "non-significant" alone is not very reliable. There is for example little difference between the p-values of 0.06 and 0.04, and making assumptions and conclusions on these minor differences seems unnecessary, when the research question involves the effect and direction of a changing parameter, rather than being binary hypothesis testing finding a yes/no answer. Confidence intervals indicate the direction and strength of the effect in question and in the present study we calculated the confidence intervals from the GLM and therefore included the odd distributions for each parameter. We made the conclusions about statistical significance from the confidence intervals and hence the result is given directly at level of the data measurement.

L302: "…surface area…". Specify dimensions of area (in Materials & methods). Since VIS/NIR was used, this would probably mean a certain volume was studied as well. State depth of NIR penetration.

Thank you for pointing this out, the depth of NIR is max 2 mm. This is now included in the manuscript.

L306: see comments to L279.

L318/Fig 4: Delete. Initial pH in muscle (t=0 h) is a key factor in this research. Unfortunately, Fig. 4 does not show these data clearly. Instead, show initial pH in a high-quality plot, same as Figs. 2 and 3 (indicate significant differences).

This figure has now been deleted and replaced with figure showing initial pH

L330: Delete "is". Add "may be". ok

L327-328: "…lower muscle pH…". This is NOT a quality problem per se. A given fish has given amount of glycogen that will be degraded to lactate. In rested fish (no exercise) this degradation occurs entirely during the early post mortem phase. In practice, however, fish are

subjected to stress during capture. A certain fraction of glycogen is consumed in vivo, as fuel for ATP. After death, the remaining glycogen is degraded. Under chilled conditions, ultimate pH is reached after about 24 h. Ultimate pH is from then onwards the same in both cases (market quality).

Lower pH is removed from this sentence

L332-332: What does "quality" imply here? See previous comments.

Quality has been replaced with residual blood

L332-343: This section should be moved to the Introduction chapter.

Thank you for this input. The section has now been moved to the introduction chapter.

L344: This is not entirely true. Excessive muscle activity is involved in both cases. What are the differences between the two cases? The fish were crowded to such an extent that increase in lactate could not come from activity caused by movement, although I do agree that the muscles are still physiological active….

The blood lactate was higher when the fish were crowded for 1 hours and more than doubled when fish were crowded for 3 hours.

This is something that should be considered before testing and further discussed here. In this particular set-up, we have at least two possibilities:

(a) excessive swimming is similar to crowding (anaerobic metabolism in white muscle)

(b) as (a) + potential pressure other fish in the cod-end + potential effect of high density where respiration (gill movement) is restricted. It would be interesting to have a few oxygen sensors in the cod-end as well as a camera set-up to monitor respiration rates.

L349-352: Yes, possibly it can affect the fish, although it is well-known (see literature) that crowded fish with ample access to DO – without restricted gill movement – have low blood and muscle pH, and high levels of lactate (see previous comment). We agree, and have included a sentence about this-

L354: refer to table

L372-374: Fillet redness…not clear what the authors refer to. Overall color or residual blood in veins? In case of overall "pink fillets", in what way can that be linked to blood? It is well known that white muscle is poorly vascularized. Residual blood in veins, or as spots, are easy to detect. By what mechanism can blood turn the entire fillet evenly pink? We have attempt to clear this in the methods chapter and the final section of the discussion.

L385-393: Delete.

Deleted

L388-389: Sometimes only "lactate" is mentioned in literature, but it is – hopefully in all cases! - implied that in reality we mean "lactate + H+". This is what happens as muscle activity is initiated: PCr is degraded to Pi + Cr, a reaction that actually consumes H+. After some seconds glycolysis sets in to maintain the ATP conc. If all reactions in the glycolytic pathway are summed up, one will find a net increase of H+. This is why we observe a drop in pH (along with increasing levels of lactate). This has been known for many decades.

This is indeed what is meant.

L395-396: Many papers do not show this effect.

Included section in discussion about this

L399-403: Delete, see also previous comments. Deleted

L405-406: Many papers do not show this effect, see previous comments.

Conclusion

Conclusion not entirely supported by obtained data. You have studied two groups of rested fish followed by crowding.

Hopefully this has been cleared up by updating the methods chapter

5

L408- Exhausted group = rested group!

L411- "…quality…"? (see previous comments)

L411-413: this is the correct conclusion from your study!

Figures

Fig. 3 – Redness scale 0-2 or 1-4 ? No dimension on y-axis for haematocrit, add (%) ok

Fig. 4 – Delete. Prepare new figure for initial pH only (same style as Figs. 2 and 3) ok

Supporting information

S1 Table

- No dimensions are given for Weight and Length. Add (g) and (cm).
- Why are air temperatures listed here? Replace with water temperatures. Water temperature is an important factor here since it determines metabolic rates (Q10). It would also be good to show water temperatures during the 2-3 weeks before the experiment was carried out (= acclimation temperature).

S1 Table is now updated in is table 1. Water temperatures are included. Air temperatures are also included as fish was netted from one tank to the swim tunnel.

S4 – Fig. 4 Check fillet redness scale! Change dimension of muscle haemoglobin axis to "g mg-1" This is now corrected

Fig. 5 – Redness scale? Corrected in methods

Figs. 6 & 8 – No dimension on haemoglobin axes… This is now corrected

Other comments

You may want to re-do your experiment with an improved experimental design. Another option would be to focus on recovery from stress (your crowding data) for up to 6 h. Consider if it makes sense to pool your two groups of rested fish.

This research is about stress physiology and effect on fillet color (or residual blood?), consequently this is what you need to discuss. Avoid further speculations on fillet quality since you do not have data to support such considerations. As already mentioned, your ms lacks a lot of essential cod data suitable for your study. Highly recommended is the excellent paper by Olsen, Sundell, Ringø et al. (2008) published in Aquaculture. It deals with different stress parameters in

blood, response and recovery times for cod. It is these issues you will want to discuss here. Check stress response times and recovery vs your sampling times. Compare your data with trawl-caught fish at sea. You need to discuss the meaning of your measured stress parameters. For cod, typical values for rested and "exhausted" cod are readily available.
